# Animal Disease in Relation to Man
*A communication to the Bristol Medico-Chirurgical Society on Wednesday, 14th March, 1951.


**Published:** 1951-10

**Authors:** A. M. G. Campbell

**Affiliations:** Physician, United Bristol Hospitals


The Bristol
Medico-Chirurgical Journal

*A Journal of the Medical Sciences for the*

West of England and South Wales

"*Scire est nescire, nisi id me
Scire alius sciret.*"

OCTOBER, 1951

### ANIMAL DISEASE IN RELATION TO MAN*

BY

A. M. G. CAMPBELL, D.M., F.R.C.P.
*Physician, United Bristol Hospitals*

Man is so much in contact with animals that it is not surprising that at times he contracts disease from them. Much work remains to be done on diseases in which the part an animal plays is still unknown. It is impossible to cover the ground of *all* diseases which either directly or indirectly are associated with animal contacts, but the following Table (I) gives an indication of some of them. Many are already familiar. Some however are not so well known, and it is with two of these in which work has been done in the Bristol area that I wish to deal: Canicola Fever, which is contracted from dogs, and toxoplasmosis, which may be contracted from a variety of animals. Before passing on to discuss these in more detail there are three points worth stressing about other infections. Many animals suffer from salmonella infections or are carriers of organisms pathogenic to man, and

* A communication to the Bristol Medico-Chirurgical Society on Wednesday, 14th March, 1951.

TABLE I

| Animal Source | Disease | Organism | Frequency | Method of Infection | Symptoms |
|---|---|---|---|---|---|
| RAT | (i) Rat bite fever | Bacillus Spirillum | Rare | Bite of rat | Undulant fever jaundice, nephritis, meningitis |
| | (ii) Leptospirosis | Spirochaete | Not uncommon | Direct (bathing) | Enteritis |
| | (iii) Salmonella | Bacillus | Not uncommon | Direct or indirect | Enteritis |
| | (iv) Plague | Bacillus | Common in Asia | Indirect, via flea or tick | Pneumonic, bubonic |
| | (v) Typhus | Virus | Common in Asia | Indirect, via flea or tick | Typhus (Typhoid-like illness) |
| MOUSE | (i) Leptospirosis murina<br>(ii) Salmonella | *See* above | | | |
| | (iii) Choriomeningitis | Virus | Rare | Indirect | Meningitis |
| DOG | (i) Tuberculosis (human) | Bacillus | Rare | Direct | Usually phthisical |
| | (ii) Canicola fever | Leptospirosis | Rare | Direct | Meningitis, Conjunctivitis |
| | (iii) Hydrophobia, Rabies | Virus | Rare except in Asia | Direct (bite) | Encephalitis, myelitis |
| | (iv) Toxoplasmosis (*see* Rabbits) | Protozoa | Rare | ? | |
| | (v) Hydatid disease | Worm | Rare except in Wales and Scotland | Eating soiled vegetables | Cysts in various organs, lung, liver, etc. |
| | (vi) Ringworm | Fungus | Rare | Direct | Skin eruption |
| BAT | (i) Hydrophobia | Virus | Rare (in Trinidad) | Bite of Vampire Bat | (*See* Dog) |
| BIRDS | (i) Psittacosis (parrots, love birds) | Virus | Rare | Direct | Pneumonia |
| | (ii) Toxoplasmosis | Protozoa | Rare | ? | (*See* Rabbit) |
| | (iii) Salmonella (duck, fowl) | Bacillus | Rare | Indirect from eggs | Enteritis |

TABLE I

| Animal Source | Disease | Organism | Frequency | Method of Infection | Symptoms |
|---|---|---|---|---|---|
| HORSE, | (i) Glanders | Bacillus | Very rare | Direct | Respiratory or dermatological |
| | (ii) Equine encephalitis | Virus | U.S.A. only | Direct: or indirect via insects | Encephalitis |
| | (iii) Anthrax (also Sheep and Cow) | Bacillus | Not uncommon | Direct or indirect | Pustule and respiratory |
| COW | (i) Brucellosis | Bacillus | Not uncommon | Direct: or indirect via milk | Undulant fever |
| | (ii) Bovine Tubercle | Bacillus | Not uncommon | Direct: or indirect via milk | All types (bone and joint) |
| | (iii) Salmonella | Bacillus | Not uncommon | Direct: or indirect via milk | Enteritis |
| | (iv) Taenia Saginata | Worm | Rare | Eating infected beef | Tapeworm infestation |
| | (v) Ringworm | Fungus | Rare | Direct | Skin or scalp infection |
| | (vi) Anthrax (*See* Horse) | | | | |
| GOAT | (i) Brucellosis | Bacillus | Common only in Malta | Direct: or indirect via milk | Undulant fever |
| SHEEP | (i) Louping ill | Virus | Rare | Direct: or indirect via milk | Meningo-encephalitis |
| | (ii) Anthrax (*See* Horse) | | | | |
| PIG | (i) Brucellosis (*see* Cow) | | | | |
| | (ii) Swine Fever | Virus | Switzerland | Direct | Influenza |
| | (iii) Taenia Solium | Worm | Not uncommon | Eating infected meat | Worm in gut or cystic (epilepsy) |
| | (iv) Trichiniasis | Worm | Common in epidemics | Eating infected meat | Cysts in muscle etc.; ac. myositis |
| HARE, RABBIT | (i) Tularaemia | Bacillus | U.S.A. and Russia | Direct: or indirect via tick | Pneumonia, rash, high fever |
| | (ii) Toxoplasmosis | Protozoa | Rare | ?Direct or indirect | Meningo-encephalitis, Eye infection |

there is considerable evidence that birds and animals thus infected may either directly or indirectly by contamination of meat in slaughtering, pass the infection on to humans: this accounts for some epidemics of food poisoning. Dogs can suffer from human tuberculosis and infect man: recent work in Sweden traced an infection of a whole family to a dog severely infected with tuberculosis, which in turn had previously contracted the infection from its former master who had died of open phthisis. Last in this category is the work done in America linking the epidemics of human encephalitis with equine encephalitis, in the transmission of which mosquitoes, tics and birds all have a part. Although equine encephalitis is not found in this country it might develop here at any time, so it is wise for us to remember this in a case of encephalitis occurring in a person working with horses.

### Canicola Fever

Canicola fever is an acute infective disease cause by the *Leptospira Canicola*, an organism of the same genus as *Leptospira Icterohaemorrhagica* which causes Weil's disease. But whereas in the latter the disease is contracted from rats, the former is a parasite of dogs. When it infects man it causes an influenza-like and meningitic illness, often complicated by eye symptoms. It is most prevalent in Flanders: but from the incidence of some twelve to fifteen cases in Bristol in the last two years, and one at least from the Dursley area, it is obviously quite frequent in the British Isles. This disease is common in dogs, particularly in large towns: about 30 to 50 per cent. of all dogs in towns have been infected, and we have demonstrated that an infected bitch can infect her own puppies. The organism is found in the urine in active cases, and two of our patients were infected by contact with urine, having had to mop up the urine of infected puppies. In one case the infection may have entered through an abrasion. We obtained evidence that dogs in the Shirehampton area were heavily infected, and at least five human cases have come from this area.

The disease usually presents as a meningitis and various neurological complications may take place such as paralysis of the cranial nerves or of the legs. Eye symptoms are common, such as mistiness of vision, iritis, diplopia and optic neuritis. The diagnosis is made by the appropriate serologicalr eactions. The disease is severe in dogs, and causes death by renal failure. Death is rare in man, but three cases are recorded, all due to uraemia. In dogs and man treatment by penicillin and streptomycin is effective. Most cases occur by direct contact with infected animals. But there is ample proof that (as in Weil's disease) infection may arise from bathing in pools or rivers contaminated by infected dogs. The preponderance of the infection in boys emphasises the greater fastidiousness of the fair sex in the choice of a bathing place. With more care in dealing with dogs' excreta, the prevention of vegetables becoming soiled, and the avoidance of dirty bathing places, this disease need never become a human problem.

### Toxoplasmosis

Toxoplasmosis has only recently been recognised in England. In animals the disease has been known since 1908 and has been found in many species. It occurs in rats, rabbits, dogs and squirrels and it is probable that animals infect man, although exactly how this occurs is unknown. The organism is a crescentic protozoon which can invade any tissue but has a predilection for the tissues of the nervous system and eye. The organism multiplies by simple binary fission and is intra-cellular, sometimes forming groups or pseudo-cysts with clusters of organisms which may degenerate to form a necrotised granulomatous mass.

Although the organism was first described by Jankû (1923) most of the credit for investigating toxoplasmosis must be shared by Wolf *et aliis* (1939) and Sabin (1941) in America. Wolf first demonstrated animal infection from human material and the congenital nature of the disease. Sabin has classified the disease into four main groups:

(i) Congenital.
(ii) Acquired disease in children.
(iii) Acquired disease in adults.
(iv) Latent disease with only pathological but no clinical findings.

The congenital cases show symptoms at birth and there may or may not be clinical evidence of the disease in the mother.

Sabin has described a triad of symptoms that he considers important in the clinical diagnosis: hydrocephalus, cerebral calcification and choroido-retinitis. It is obvious that only one of these symptoms may be present and the case may still be one of toxoplasmosis. Callahan (1946) found in St. Louis in 10,000 autopsies five cases of infantile toxoplasmosis which previously had passed unrecognised; this sort of incidence is probably true of other cities. Apart from the clinical recognition of the disease diagnosis rests on the finding of the organism (which is extremely difficult) and on demonstration of antibodies in the patient's serum. The value of the serological and pathological tests in this condition have been fully reviewed by Cathie and Dudgeon (1949). Evidence is now accumulating that many persons show immune bodies to toxoplasmosis, and Sabin (1950) found figures as high as between 10 and 60 per cent. of positive serological tests in " normal " people in certain American cities. Before a diagnosis of toxoplasmosis can be reached it is necessary to weigh the clinical and pathological evidence most carefully.

*The Disease in Adults.* Sabin (1942) described an acquired form of the disease in older children, stressing the occurrence of acute encephalitis. As early as 1914 Castellani had claimed to have found an organism resembling toxoplasmosis in a Cingalese boy of 14 years, dying of an unknown fever with splenomegaly and anaemia. Pinkerton and Henderson (1941) described an acute illness in adults which resembled typhus fever, in which there was a macular rash, pyrexia, and pulmonary signs; and in whom toxoplasma were demonstrated at the post-mortem. Their cases showed a resemblance to the Rickettsial diseases, and in both cases was a history of a bite by a tick. In one of their patients the disease was successfully transmitted to guinea-pigs. Guimaraes (1943) described a young Brazilian negro who died of an acute meningo-encephalo-myelitis at the age of eighteen. At post-mortem the patient showed extensive inflammatory areas throughout the brain from which toxoplasma were isolated.

Syverton and Slavin (1946) described an illness in a 65-year-old tailor with fever, diarrhoea and joint pains, a macular rash and a persistent eosinophilia of from 25 to 45 per cent. Biopsy material from this case showed it to be a case of toxoplasmosis. This man had had an unexplained rash twenty-five years previously which may well have been a manifestation of toxoplasma infection. It suggested that the disease might remain latent for many years, and that, as in the cases we propose to record, fluctuations of the rash and suffusion of the eyes may have been an allergic reaction to toxoplasma antigen. Brennan (1950) has suggested that increase in the severity of the rash coincides with the release of new toxoplasma antigen from an internal focus. Binkhorst (1949) records in the mother of one of his cases the occurrence of a rash during her pregnancy which may have been due to toxoplasmosis, and the mother of the case recorded by Wyllie (1950) had a similar rash.

The familial side of this disease has not been adequately studied. The pathological tests have indicated that in affected infants and children the mother has often shown either clinical signs of choroido-retinitis, which were previously undetected, or positive antibody reactions. Zuelzer (1949) has recorded the case of twins who suffered from the disease, and Robson (1950) has studied the case of a father and child who had both had a fever and encephalomyelitis; but the father's case history is somewhat indefinite and his case cannot be definitely regarded as one of toxoplasmosis, although several members of this family had choroido-retinitis and positive serological tests.

In the Bristol area two families with toxoplasmosis have been investigated. The signs and symptoms of the first family are fully outlined in Table II, and this family illustrates very well the adult type of the disease. The second family consisted of a clinically normal, but pathologically positive, mother whose first child is in the Blind School with hydrocephalus, choroido-retinitis, optic atrophy and dementia: her second child died three days after birth of jaundice, probably from toxoplasmosis, and a third child, now three months old, showed positive serological tests at birth. This case is still under investigation.

To return to the first family, which has many unusual features. The picture of these cases can be compared to the behaviour of congenital syphilis, and there is evidence of transmission through three generations. It is impossible to say with certainty whether the disease originated in the mother of the original patient, or his grandmother. This lady has shown one positive antibody test only. The infection has remained present in a sub-acute form in

TABLE II (First Family)

*Comparison of three*

| | *Mrs. C.* | *R.C., son* | *Mrs. G., daughter* |
|---|---|---|---|
| Headache and sickness ... | + since age of 5 | ++ since age of 8 Very acute recently | ++ since age of 14 |
| Rash intermittent)... ... | ++ | ++ | ++ |
| Suffusion of conjunctiva ... | ++ | ++ | ++ |
| Pyrexia ... ... ... | – | + | – |
| Saddle-back Nose ... | + | + | + |
| Claw Feet ... ... | – | + | + |
| Joint Pains ... ... | + | + | + |
| Cough ... ... ... | + | – | – |
| Hydrocephalus ... ... | – | + | + |
| Intra-cerebral calcification | – | – | – |
| Micro-ophthalmos ... | + | – | – |
| Micro-cornea ... ... | + | – | – |
| Choroido-retinitis ... | +++ macular and peripheral | + slight peripheral | ++ peripheral |
| Optic atrophy ... ... | +++ | ++ | + |
| Papilloedema ... ... | – | ++ | + |

three members of one family, and possibly four, for many years, causing chronic ill health, headaches and the symptoms shown in Table II. It appears that a state of chronic toxoplasma infection can exist in adults where the antibody response is insufficient to deal with the infection. In most persons infected the antibody response is obviously adequate, with the exception of the foetus in utero which is very liable to a severe and fatal infection. As yet we have no knowledge of how man may acquire the infection: but it is becoming increasingly evident that dogs may become infected and their proximity to man makes them a most likely source of human infection. Recently the disease has been transmitted to guinea pigs by vaginal inoculation.

TABLE II (First Family)

*members most affected*

| | *Mrs. C.* | *R.C., son* | *Mrs. G., daughter* |
|---|---|---|---|
| Opaque nerve fibres ... | — | + | + |
| Nerve deafness ... ... | +++ | + | ++ |
| Meningo-encephalitis ... | ++ | ++ | + |
| Spasticity of legs ... ... | + | + | — |
| Extensor plantar reflex ... | + | + bilateral | + left only |
| Splenomegaly ... ... | + | — | + temporary only |
| Wassermann Reaction ...<br>Blood ...<br>C.S.F. ... | —<br>—<br>— | —<br>—<br>— | —<br>—<br>— |
| C.S.F. Pressure ... ...<br>Pleocytosis... ...<br>Protein, per cent. | Normal<br>20–30<br>110–160 mg. | 300 mm.<br>80–190<br>70–95 mg. | Normal<br>20–30<br>20–65 mg. |
| Eosiniphilia<br>Blood: per cent. ...<br>C.S.F.: per cent. | <br>4–8<br>Unknown | <br>10<br>5–10 | <br>4<br>6 |
| Cytoplasm modifying test... | + at 1/32 | + at 1/128 | + at 1/32 |
| Complement fixation test... | + at 1/8<br>+ at 1/16 | Eight times<br>+ at 1/16<br>+ at 1/8 | + at 1/16 |
| Toxoplasmin Skin test ... | Not performed | + 24 hours<br>+ 48 hours | + 24 hours<br>+ 48 hours |

It may well be that certain persons are more susceptible to infection than others, and this would explain the large number of positive antibody tests in apparently healthy persons. The persistence of proliferation of toxoplasma in the eye and central nervous system may be explained by the poor diffusion of serum antibody into these tissues. The family recorded must have had a poor antibody response. As has already been suggested, the continued release of antigen accounts for the rash, uveitis and eosinophilic response seen in the patients.

The case recorded by Brennan (1949) resembles this family most closely in that a man of thirty was found to have the same generalised cutaneous eruption, choroido-retinitis and eosinophilia, with persistent but fluctuating headaches, with positive antibody tests and positive intra-dermal tests for toxoplasma antigen. This patient also had an intermittent fever, as shown by the patient, R.C. The eosinophilia was 4 per cent. This patient appeared to improve on sulphonamides, in the same way as R.C., but relapsed when they had to be discontinued due to haematuria.

The nerve deafness found in all these case has not been previously recorded in toxoplasmosis, and it is important for aural specialists to bear toxoplasmosis in mind when examining cases of nerve deafness in children and young adults. The choroido-retinitis shown by these cases is much more slight and peripheral than the gross lesions seen in some cases where there is a large area of choroido-retinitis near the macula. The pepper-and-salt appearance of small lesions in these cases was much more marked in Mrs. C. than in her children. Therapy presents a most difficult problem as most drugs, with the possible exception of sulphonamides, have little effect on the organism. Frenkel (1949) has reported some improvement in retinal lesions with graduated doses of toxoplasma antigen, and it might be worthy of trial in the case R.C.: but we fear that many of the changes in these chronic cases are irreversible. The poor results of therapy are strictly in accord with the experience of other workers.

In conclusion, this paper has merely touched on a fascinating problem in epidemiology and public health. Such questions as whether " swayback " is related to disseminated sclerosis, or whether there is an animal vector or reservoir of the poliomyelitic virus, remain unsolved. Nor do I advocate our casting away our animal friends: rather that we remember we can get disease from a sick animal or a sick bird, and we should take the same precautions with them to avoid infection as we would take in a case of a known human infectious disease, particularly where food and excreta are concerned. We should as a profession have more contact and discussion with veterinary surgeons about prevalent animal epidemics in relation to human disease. The two professions can learn a great deal from each other, and in the past this fact has been neglected. It may be of interest that a good observation by a Weston-super-Mare doctor has, since this paper was read, shown a child with paratyphoid B. had become infected by her pet tortoise.

### REFERENCES

1. Binkhorst, C. D. (1948).—*Toxoplasmosis*, Kroese, Leiden.
2. Brennan, A. J., Brown, T., Warner, J. and Vranian, G. (1949).—*Amer. Journ. Med.*, **7**, 431.
3. Campbell, A. M. G., and Broom, J. C., *et al.* (1950).—*B.M.Jl.*, 1950, i, 336.
4. Campbell, A. M. G., *et al.* (1947).—*Brain*, **70**, 50.
5. Campbell, A. M. G. and Clifton, F. (1950).—*Ibid*, **73**, iii, 281.
6. Castellani, A. (1914).—*Jl. Trop. Med.*, **17**, 113.
7. Cathie, I. A. B. and Dudgeon, J. A. (1949).—*Journ. of Clin. Path.*, **2**, 259.
8. Frenkel, J. K. (1949).—*Jl. A.M.A.*, **85**, 140, 369.
9. Guimaraes, F. N. (1943).—*Mem. do Inst. Oswaldo.*, Cruz., **38**, 257.
10. Jacoby, N. M. and Sagorin, L. (1948).—*Lancet*, 1948, ii, 926.
11. Jankû, J. (1924).—*Z. Bl. F. die Ges. Ophthalm.*, **12**, 112.
12. Pinkerton, H. and Henderson, R. G. (1941).—*Jl. A.M.A.*, **116**, 807.
13. Sabin, A. B. (1941).—*Ibid.*, **116**, 801.
14. Syverton, J. F. and Slavin, H. B. (1946).—*Ibid.*, **131**, 12, 957.
15. Wolf, A., Cowan, D. and Paige, B. H. (1939).—*Science*, **89**, 226.
16. Wyllie, W. G., Fisher, H. J. W. and Cathie, I. A. B. (1950).—*Quart. Jl. Med.*, **19**, 73, 57.
17. Zuelzer, W. W. (1944), *Arch. Path.*, **38**, 1.

